# Phosphate of Lime in Its Relation to the Nutrition of Animals, and the Mortality of Children

**Published:** 1853-03

**Authors:** 


*Phosphate of Lime in its Relation to the Nutrition of Animals, and the Mortality of Children.*—Dr. Mourier addressed a memoir on this subject to the French Academy of Sciences, in July last, in which he states that the principal object of phosphate of lime in the system is not, as is commonly supposed, the formation and nourishment of the bones; but that its more special action is to produce and keep up vital irritability, and that it is found in animals in a definite quality, but varying according to their temperature, youth and vital activity. He says that in cities especially, the fœtus and infant seldom obtain the quantity necessary for their developement and existence; hence, this is evidently one of the causes of the very great mortality of children, especially in cities.—*Archives Génér.*, Sept. 1852.

